# Surface Modification of FeCoNiCr Medium-Entropy Alloy (MEA) Using Octadecyltrichlorosilane and Atmospheric-Pressure Plasma Jet

**DOI:** 10.3390/polym12040788

**Published:** 2020-04-02

**Authors:** Pei-Yu Cheng, Nian-Hu Lu, Yi-Sheng Lu, Chih-Hsuan Chen, Yueh-Lien Lee, Jian-Zhang Chen

**Affiliations:** 1 Graduate Institute of Applied Mechanics, National Taiwan University, Taipei City 10617, Taiwan; r07543033@ntu.edu.tw; 2 Advanced Research Center for Green Materials Science and Technology, National Taiwan University, Taipei City 10617, Taiwan; 3 Department of Mechanical Engineering, National Taiwan University, Taipei City 10617, Taiwan; f06522712@ntu.edu.tw; 4 Department of Engineering Science and Ocean Engineering, National Taiwan University, Taipei City 10617, Taiwan; r06525019@ntu.edu.tw

**Keywords:** atmospheric-pressure plasma jet, surface treatment, hydrophobicity, medium entropy alloy, oxidation, octadecyltrichlorosilane

## Abstract

Surface condition and corrosion resistance are major concerns when metallic materials are going to be utilized for applications. In this study, FeCoNiCr medium-entropy alloy (MEA) is first treated with a nitrogen atmospheric-pressure plasma jet (APPJ) and then coated with octadecyltrichlorosilane (OTS) for the surface modification. The hydrophobicity of the FeCoNiCr MEA was effectively improved by OTS-coating treatment, APPJ treatment, or the combination of both treatments (OTS-coated APPJ-treated), which increased the water contact angle from 54.49° of the bare MEA to 70.56°, 93.94°, and 88.42°, respectively. Potentiodynamic polarization and electrochemical impedance spectroscopy tests demonstrate that the APPJ-treated FeCoNiCr MEA exhibits the best anti-corrosion properties. X-ray photoelectron spectroscopy reveals that APPJ treatment at 700 °C oxidizes all the alloying elements in the FeCoNiCr MEA, which demonstrates that a short APPJ treatment of two-minute is effective in forming a metal oxide layer on the surface to improve the corrosion resistance of FeCoNiCr MEA. These results provide a convenient and rapid method for improving surface properties of FeCoNiCr MEA.

## 1. Introduction

Medium-entropy alloys (MEAs) contain multiple principal elements with high mixing entropy for stabilization in a disordered solid solution state [1,2,3,4]. In particular, FeCoNiCrMn-based materials have attracted attention for their excellent combination of properties such as good fracture resistance, high tensile strength and ductility, excellent cryogenic properties, and superplasticity. This alloy family is based on face-centered cubic (fcc) FeCoNiCr solid solution [5]. Due to the high potential for applications, various methods were introduced to further improve the properties of FeCoNiCr MEA, including elemental alloying [6,7], precipitation hardening [8,9], and processing [10,11]. To utilize MEAs into real applications, their surface properties and corrosion resistance are critical concerns. Because MEAs contain multiple principal elements, their oxidation and corrosion behaviors are usually quite complicated and have been investigated widely [4,12,13,14].

Octadecyltrichlorosilane (OTS) is a frequently used chemical for modifying the hydrophilic –OH attached surface into a hydrophobic surface [15,16,17]. By well controlling the immersion time, water content, and silane concentration, a self-assembled monolayer (SAM) could be formed on the oxide surface [18,19,20]. The quality of the oxide underneath also influences the quality of the grown SAM [18]. A silane coating has been used for improving the anticorrosion properties of metals and alloys [21,22]. Typically, MEAs have native oxides on the surface, and therefore, it is possible to modify the surface of a MEA with silane-based molecules. Because the quality of the oxide underneath could influence the quality of the follow-up deposited SAM, an atmospheric-pressure plasma jet (APPJ) is employed to oxidize FeCoNiCr MEAs prior to OTS coating.

An atmospheric-pressure plasma (APP) can be operated at regular pressure without using vacuum systems that demand routine maintenance. Several techniques have been used to develop a stable APP, including transfer arc, corona, dielectric barrier discharge, and APPJ [23,24,25,26,27]. Various electrode configurations and excitation methods generate APPs with different gas and electron temperatures [28]. APP with low gas temperature has been used for applications in biomedicine, food processing, and agriculture [29,30]. An APP with an intermediate gas temperature (of the order of several hundred degrees Celcius) can be used for rapid materials processing by taking advantage of the synergetic effect of heat and reactive plasma species [28,31,32,33,34,35,36,37,38,39,40,41]. APP has been used to oxidize the AA6061-T6 aluminum alloy surface for strong and durable adhesive bonding applications [42]. An APPJ has also been used for generating a corrosion protection for a copper surface [43]. An APP polymerized fluorine-rich coating demonstrated to enhance corrosion resistance and hemocompatibility for a biomedical NiTi alloy [44].

In this study, we experimentally test a nitrogen APPJ with working temperature of ~700 °C to oxidize the surface of FeCoNiCr MEAs. Then, OTS is used to modify the surface. With these surface modification treatments, the hydrophobicity, corrosion resistance and surface bonding conditions of the FeCoNiCr MEA are investigated and analyzed to understand the improvement of surface properties.

## 2. Experimental Detail

### 2.1. Preparation of FeCoNiCr MEA

The equiatomic FeCoNiCr MEA was prepared by vacuum arc melting under a high-purity Ar atmosphere. High-purity (>99.9 wt %) Fe, Co, Ni, and Cr elements were used as raw materials. Before melting the FeCoNiCr ingot, a pure Ti ingot was melted two times to reduce the oxygen content in the chamber. To improve the chemical homogeneity of the material, the FeCoNiCr ingot was flipped and remelted six times. The ingot was homogenized at 1200 °C for 24 h under Ar atmosphere in a tubular furnace and then subjected to furnace cooling. The homogenized ingot was then cold-rolled to an 80% reduction in thickness. The cold-rolled plate was then annealed at 900 °C for 1 h and then cut into 2 × 2 cm samples using a diamond saw.

### 2.2. Pretreatment of FeCoNiCr MEA before APPJ Treatment

First, the FeCoNiCr MEA specimens were mechanically abraded using sand paper with mesh number up to P2000. Next, the specimens were sequentially ultrasonicated in deionized water, acetone, and isopropanol; each ultrasonication was performed for 15 min. After ultrasonication, the specimens were blow-dried using a N_2_ gun.

### 2.3. APPJ Treatment of FeCoNiCr MEA

**Figure 1:**
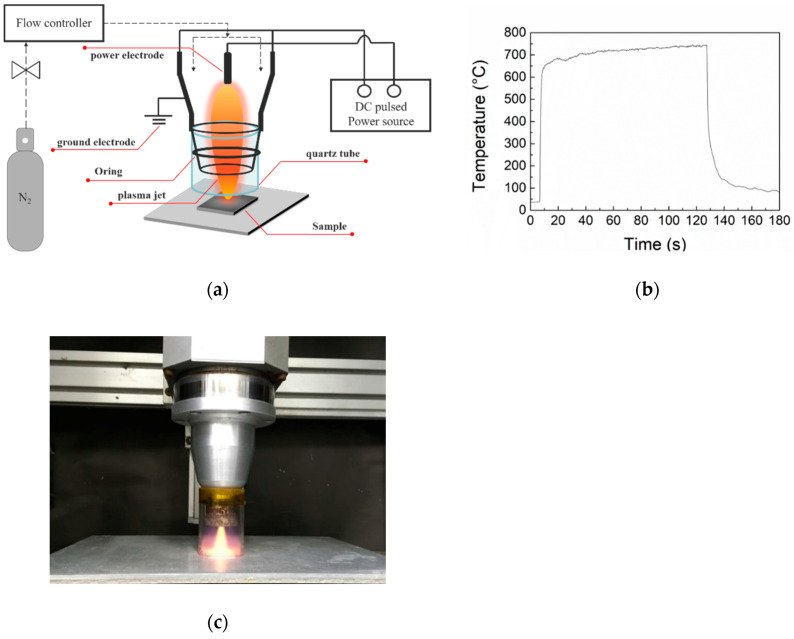
(**a**) Schematic of atmospheric-pressure plasma jet (APPJ) setup; (**b**) working temperature evolution of APPJ; (**c**) photograph of APPJ during processing.

Figure 1a shows the APPJ setup used in this study. The voltage, frequency, and duty cycle of the APPJ were 275 V, 25 kHz, and 17.5%, respectively. The N_2_ flow rate was fixed as 34 standard liters per minute (slm). Reduce the air-quenching effect from ambient air, a quartz tube with length of 4.5 cm and internal diameter of 3 cm was installed downstream of the plasma jet exit. This arrangement can increase the plasma jet length and expand the plasma influential zone. The temperature at the sample surface was monitored using a K-type thermocouple. Figure 1b shows the temperature evolution. The temperature plateaued at ~700 °C. The APPJ treatment lasted for 2 min. Figure 1c shows the photograph of APPJ during processing.

### 2.4. OTS Coating

**Figure 2:**
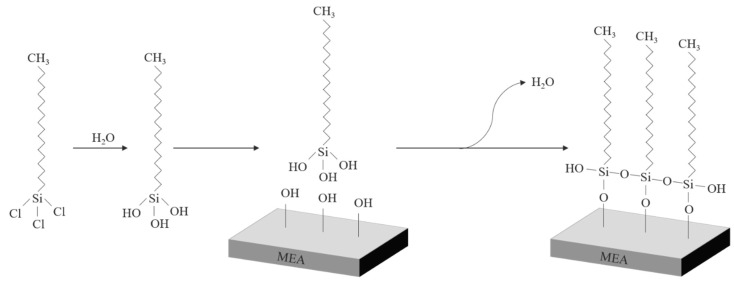
Schematic diagram of octadecyltrichlorosilane (OTS) self-assembly reaction process.

The OTS coating is performed using a solution process. First, 4 µL of OTS (95%, Acros Organics, Waltham, MA, USA) was injected into 10 mL of n-dodecane (99+%, Alfa Aesar, Ward Hill, MA, USA) in a beaker. FeCoNiCr MEA specimens with/without APPJ treatment were immersed in the solution with ultrasonication for 15 min. Figure 2 shows the OTS self-assembly reaction process. It is generally accepted that OTS molecules are either chemisorbed or physisorbed on the oxide surface with some molecules forming short-range cross-linked structures [15].

### 2.5. Materials Characterization

The water contact angle was measured using a goniometer (Model 100SB, Sindatek Instruments Co., Ltd., New Taipei City, Taiwan). An electrochemical workstation (Metrohm Autolab, PGSTAT204, Ionenstrasse, Switzerland) was used to evaluate the corrosion resistance behavior of specimens in a 3.5 wt % NaCl through potentiodynamic polarization tests and electrochemical impedance spectroscopy (EIS) tests. A standard three-electrode system was used for electrochemical measurements. The specimen is the working electrode, a platinum wire and Ag-AgCl is used as the counter and reference electrodes, respectively. The potentiodynamic polarization test is performed starting from a potential from −0.5 to 1 mV v.s. open circuit potential (OCP) at a scan rate of 1 mV/s. The EIS plots were acquired at OCP in a frequency range of 10^5^–10^−2^ Hz by using an alternating current with the amplitude of 10 mV (rms). The surface chemical bonding status was investigated using X-ray photoelectron spectrometry (XPS, VGS Thermo Scientific K-Alpha, Waltham, MA, USA). The binding energy (BE) was calibrated with a C1s peak at 284.8 eV. The crystallinity was inspected using an X-ray diffractometer (XRD, Bruker D8 DISCOVER SSS Multi-Function High-Power X-Ray Diffractometer, Billerica, MA, USA). The surface morphology was inspected using a scanning electron microscope (SEM, JOEL JSM-7800 Prime, Tokyo, Japan).

## 3. Results and Discussion

**Figure 3:**
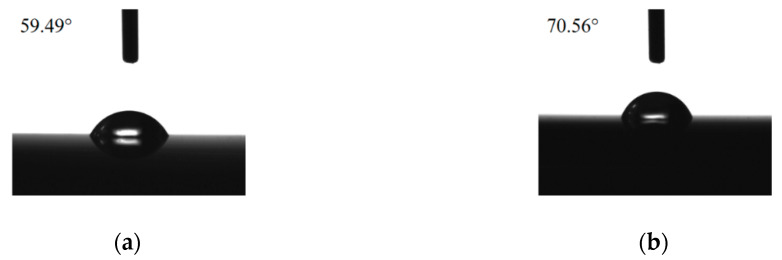

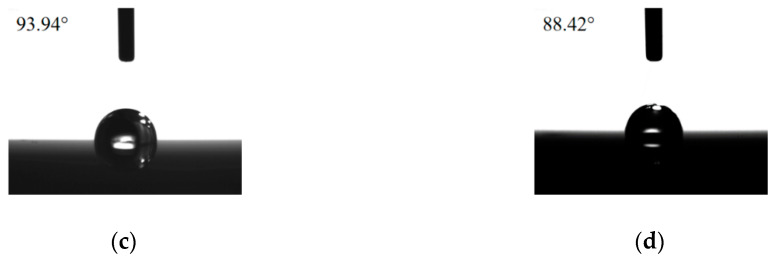
Water contact angles of (**a**) FeCoNiCr medium-entropy alloy (MEA), (**b**) APPJ-treated FeCoNiCr MEA, (**c**) OTS-coated APPJ-treated FeCoNiCr MEA, and (**d**) OTS-coated FeCoNiCr MEA.

Figure 3a shows the water contact angle (59.49°) for FeCoNiCr MEA without OTS and APPJ processing. After APPJ processing, the water contact angle increased to 70.56°, as shown in Figure 3b, possibly owing to the oxidation of FeCoNiCr MEA. Figure 3c shows the water contact angle after APPJ and OTS processing. The water contact angle is 93.94°. For comparison, we also performed OTS-coating on FeCoNiCr MEA without APPJ treatment; the water contact angle is 88.42°, as shown in Figure 3d. OTS coating significantly increased the water contact angle and hydrophobicity, indicating successful coating of OTS on FeCoNiCr MEA. With APPJ processing followed by OTS coating, the water contact angle is the largest, possibly owing to the formation of surface oxides that could facilitate the follow-up OTS coating. With a better quality of the OTS coating layer, the hydrophobicity of the FeCoNiCr surface is further improved.

**Figure 4:**
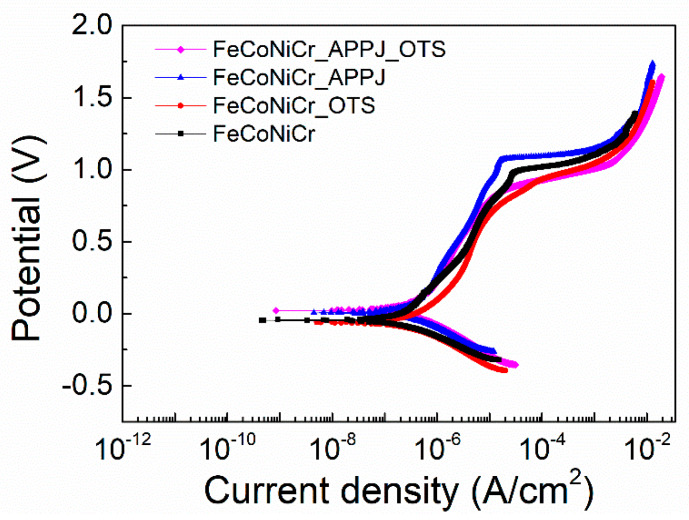
Potentiodynamic polarization test curves of bare, OTS-coated, APPJ-treated, and OTS-coated APPJ-treated FeCoNiCr MEAs.

Figure 4 shows the potentiodynamic polarization curves. FeCoNiCr, FeCoNiCr_OTS, FeCoNiCr_APPJ, and FeCoNiCr_APPJ_OTS represent bare, OTS-coated, APPJ-treated, and OTS-coated APPJ-treated FeCoNiCr MEAs, respectively. Table 1 lists the corresponding corrosion potential (Ecorr), corrosion current density (Icorr), pitting potential (Epit), and passive region (Epit-Ecorr) values determined with methods described in [45,46]. As shown from the table, APPJ treatment can increase pitting potential and passive region range of FeCoNiCr MEA. In addition, APPJ treatment also reduces the current density in the passive region. This enhancement in anti-corrosion properties can be attributed to the formation of metal oxides on the surface of FeCoNiCr MEA. However, the lower pitting potential and narrower passive region were observed in OTS-coated APPJ-treated FeCoNiCr MEA sample. It is plausible that the ultrasonication during OTS coating may produce defects on part of the loosely grown oxides during APPJ treatment. Nevertheless, APPJ treatment increases Ecorr from −0.045 V to 0.006 V. OTS coating does not drastically affect the corrosion potential and the current density in the passivation area compared with the bare and APPJ-treated MEAs. The OTS coating treatment after APPJ treatment improves the Ecorr further to 0.022 V, however, the passive region drops to a level worse than the one without any treatment. Following the analyses, although the OTS coating increases the water contact angle, as shown in Figure 3, it seems not to be a suitable protective layer to prevent corrosion. In the end, the APPJ-treated MEA shows the best anti-corrosion performance based on the indicators of the pitting potential and the width of the passive region range. Figure S1 shows the XRD results, and Table S1 summarized corresponding crystallinity information obtained from XRD results in Figure S1. After the APPJ treatment, the grain size slightly increased because of the thermal effect. The relation between crystal size and corrosion may vary among different materials. In one particular case, the previous report indicates that grain size of 304L austenitic stainless steel has no effect on pitting potential [45].

**Figure 5:**
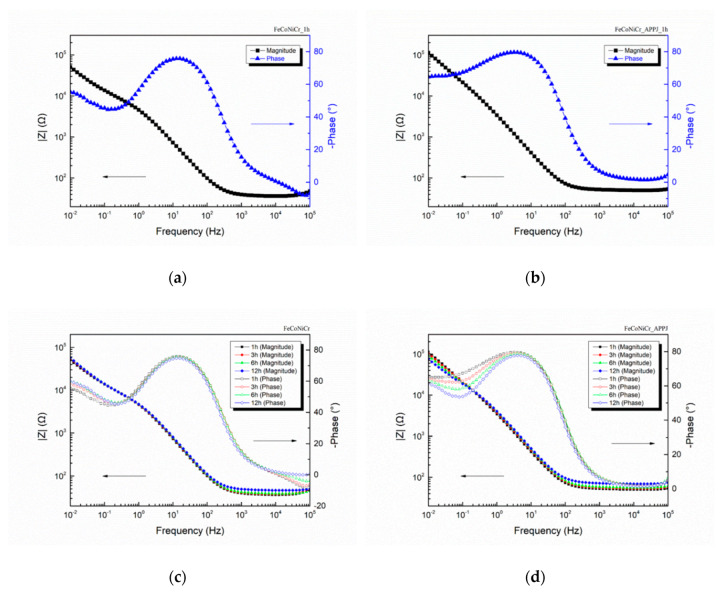
Bode magnitude and Bode phase plots of (**a**) bare MEA after 1 h, (**b**) APPJ-treated MEA after 1h, (**c**) bare MEA for 12 h, and (**d**) APPJ-treated MEA for 12 h.

From potentiodynamic polarization curves, APPJ treated-MEA shows better corrosion-resistant properties. For clarity, hereafter we compare data with bare MEA and APPJ-treated MEA. Figure 5 shows the results of the EIS plots of bare and APPJ-treated MEAs. Figure 5a,b show the comparative results of Bode magnitude and Bode phase plots from two types of samples after first hour of immersion. The evolution of EIS Bode plots from two types of samples during immersion time up to 12 h is reported in Figure 5c,d. The EIS measurement results for all cases (bare, APPJ-treated, OTS-coated, OTS-coated APPJ-treated MEAs) are listed in the Supplementary Information.

As can be seen in Figure 5a,b, the impedance value at low frequency (|Z|0.01Hz) of APPJ-treated MEA (112995Ω) was higher than that of bare MEA (50704Ω) at the beginning of the EIS measurement. Although a slight decrease of |Z|0.01Hz for APPJ-treated FeCoNiCr MEA was observed as the immersion time increased, as shown in Figure 5d, APPJ-treated FeCoNiCr MEA still exhibited a higher |Z|0.01Hz value compared with that obtained from bare FeCoNiCr MEA, suggesting that APPJ treatment can enhance the anti-corrosion properties of FeCoNiCr MEA. In addition, Figures S2–S5 show the details of EIS plots for each specimen.

**Figure 6:**
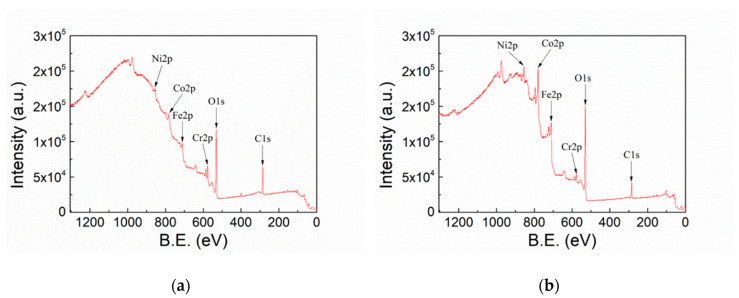
XPS survey scan spectra of (**a**) FeCoNiCr and (**b**) APPJ-treated FeCoNiCr.

Figure 6 shows the XPS survey scan spectra of FeCoNiCr and APPJ-treated FeCoNiCr MEAs. Table 2 lists the atomic contents analyzed from Figure 6. Carbon content decreased [43] and oxygen content increased after APPJ processing, indicating the removal of organic contaminants and oxidation of FeCoNiCr MEA.

**Figure 7:**
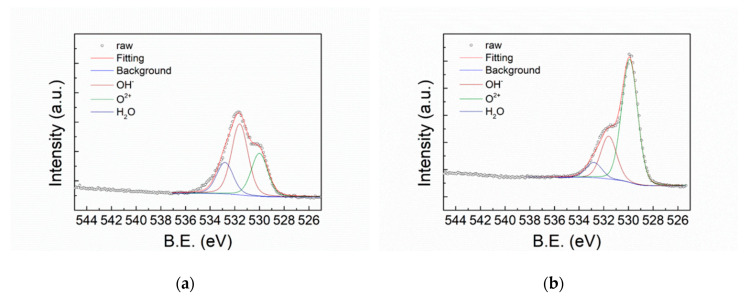
XPS O1s spectra of (**a**) FeCoNiCr and (**b**) APPJ-treated FeCoNiCr MEAs.

**Figure 8:**
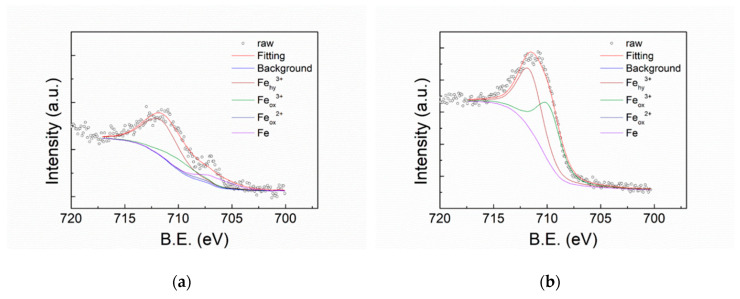
XPS Fe2p spectra of (**a**) FeCoNiCr and (**b**) APPJ-treated FeCoNiCr MEAs.

**Figure 9:**
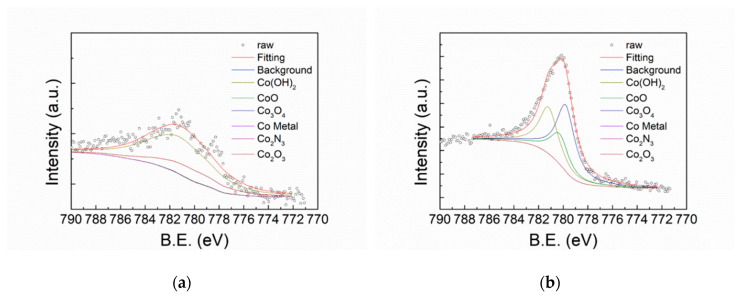
XPS Co2p spectra of (**a**) FeCoNiCr and (**b**) APPJ-treated FeCoNiCr MEAs.

**Figure 10:**
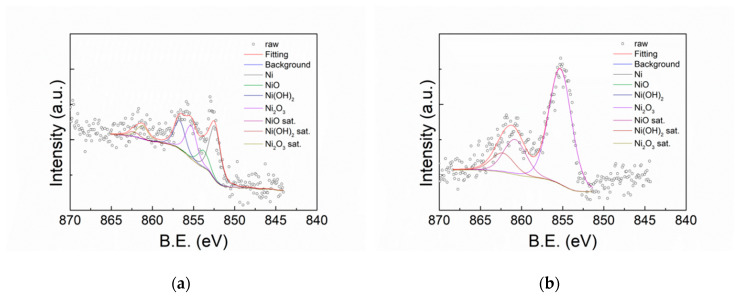
XPS Ni2p spectra of (**a**) FeCoNiCr and (**b**) APPJ-treated FeCoNiCr MEAs.

**Figure 11:**
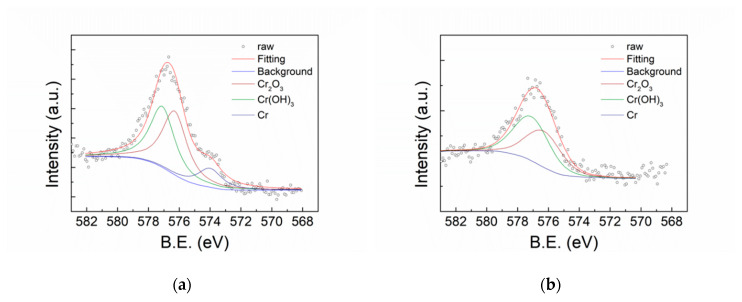
XPS Cr2p spectra of (**a**) FeCoNiCr and (**b**) APPJ-treated FeCoNiCr MEAs.

Figure 7 shows the XPS O1s spectra of FeCoNiCr and APPJ-treated FeCoNiCr MEAs. Deconvoluted peaks represent O_2+_ (530 eV), OH (531.6 eV), and H_2_O (532.8 eV) [4]. Table 3 lists the areal atomic ratio of these contents. After APPJ treatment, the overall peak intensity significantly increased, suggesting the occurrence of oxidation. Figure 8 shows the XPS Fe2p spectra that can be deconvoluted into four components, metallic Fe (706.6 eV), Fe_ox_^2+^ (708.2 eV), Fe_ox_^3+^ (709.8 eV), and Fe_hy_^3+^ (711.6 eV) [4,46]. Table 4 lists the ratio of these four components. Metallic Fe (Fe^0^) content significantly decreased from 15.10% to 0% and Fe^2+^ decreased from 3.4% to 0%, whereas Fe^3+^ increased from 19.06% to 49.23%; this strongly suggests the oxidation of the Fe component by APPJ treatment. Fe is oxidized into Fe^3+^ state after APPJ processing. Figure 9 shows the XPS Co2p spectra and Table 5 lists the ratio of deconvoluted components, metallic Co (777.5 eV), Co_3_O_4_ (779.8 eV), CoO (780.3 eV), Co(OH)_2_ (781.2 eV), Co_2_O_3_ (780.4 eV), and Co_2_N_3_ (778.1 eV) [4,47,48]. No metallic Co was detected even without APPJ treatment, implying high oxidization proportion of Co on the surface. After APPJ treatment, alteration of cobalt oxide status was noted. The whole surface was in the oxidized state for Co. No Co nitridation was noted with nitrogen APPJ treatment. Figure 10 shows the XPS Ni2p spectra that can be deconvoluted into Ni (852.4 eV), NiO (853.8 eV), Ni(OH)_2_ (856.7 eV), Ni_2_O_3_ (855.3 eV), NiO_sat._ (860.8 eV), Ni(OH)_2 sat._ (862.3 eV), and Ni_2_O_3 sat._ (861.2 eV) [4,49]. Table 6 lists the component ratio. After APPJ processing, all Ni components including large portions of metallic Ni and NiO and a small amount of Ni(OH)_2_ were oxidized into Ni_2_O_3_. Before APPJ treatment, the surface of FeCoNiCr MEA contained 16.59% metallic Ni and 44.36% NiO. All these components were completely oxidized into Ni_2_O_3_. Figure 11 shows the XPS Cr2p spectra that can be deconvoluted into metallic Cr (574 eV), Cr_2_O_3_ (576.3 eV), and Cr(OH)_3_ (577.1 eV) [4,50,51]. Table 7 lists the ratio of each component. Before APPJ treatment, 13.22% metallic Cr was seen on the surface. After APPJ treatment, the metallic Cr content reduced to 0% and the Cr(OH)_3_ component increased. This also indicates oxidation of Cr by APPJ processing. Overall, all metal components in FeCoNiCr MEA were oxidized by nitrogen APPJ treatment because of the involvement of oxygen from ambient air in APPJ processing. The APPJ temperature was set as 700 °C; in this high-temperature environment, MEA oxidation occurred easily. The oxidations of all the metallic components in the FeCoNiCr MEA formed effective surface oxide layer which contributed to the better follow-up OTS treatment and the better corrosion resistance. Experimental results demonstrate that APPJ treatment is a convenient and economic method to improve the anti-corrosion properties of FeCoNiCr MEA. Furthermore, Figure S6 shows the SEM images of specimens of bare, APPJ-treated, OTS-coated APPJ-treated and OTS-coated MEAs. No apparent morphology difference is noted.

## 4. Summary

We use a nitrogen APPJ and OTS coating for the surface modification of FeCoNiCr MEA. A short nitrogen APPJ treatment for 2 min at 700 °C oxidized FeCoNiCr MEA. The metal oxide layer resulted from the APPJ treatment not only increases the passivation region and pitting potential but also decreases the current density of the passivation area. An OTS coating improves the hydrophobicity but narrows down the passivation region. Nevertheless, an OTS coating does not drastically influence the corrosion potential, the corrosion rate, and the current density in the passivation area. OTS-coated APPJ-treated FeCoNiCr MEA shows the highest hydrophobicity with water contact angle of 93.94°, however, APPJ-treated FeCoNiCr MEA shows the best anti-corrosion property. The APPJ and OTS coating methods introduced in this study provide convenient and economic surface modifications to improve the corrosion resistance and surface hydrophobicity of FeCoNiCr MEA.

## Figures and Tables

**Table 1:** E_corr_ and I_corr_ for bare, OTS-coated, APPJ-treated, and OTS-coated APPJ-treated FeCoNiCr MEAs. Three measurements were performed.

Sample	E_corr_ (V)	I_corr_ (µA/cm^2^)	E_pit_ (V)	ΔE = E_pit_ − E_corr_ (V)
FeCoNiCr	−0.069 ± 0.013	53.2 ± 32.3	0.997 ± 0.020	1.066
FeCoNiCr_APPJ	−0.046 ±0.029	60.5 ± 31.9	1.068 ± 0.005	1.114
FeCoNiCr_OTS	−0.064 ± 0.018	51.2 ± 11.3	0.772 ± 0.011	0.836
FeCoNiCr_APPJ_OTS	−0.021 ± 0.022	54.5 ± 0.8	0.826 ± 0.004	0.847

**Table 2:** Elemental ratios from XPS survey scan in Figure 6.

	C1s (at%)	O1s (at%)	N1s (at%)	Cr2p (at%)	Fe2p (at%)	Ni2p (at%)	Co2p (at%)
FeCoNiCr	42.25%	39.68%	4.62%	4.79%	2.94%	2.59%	3.12%
FeCoNiCr_APPJ	16.1%	49.9%	1.47%	2.41%	13.09%	4.21%	12.81%

**Table 3:** XPS O1s deconvoluted peak areal ratios from XPS spectra shown in Figure 7.

	O^2+^ (at%)	OH^−^ (at%)	H_2_O (at%)
FeCoNiCr	29.57%	48.84%	21.60%
FeCoNiCr_APPJ	67.97%	23.65%	8.38%

**Table 4:** XPS Fe2p deconvoluted peak areal ratios from XPS spectra shown in Figure 8.

	Fe (at%)	Fe_ox_^2+^ (at%)	Fe_ox_^3+^ (at%)	Fe_hy_^3+^ (at%)
FeCoNiCr	15.10%	3.40%	19.06%	62.44%
FeCoNiCr_APPJ	0.00%	0.00%	49.23%	50.77%^1^

**Table 5:** XPS Co2p deconvoluted peak areal ratios from XPS spectra shown in Figure 9.

	Co Metal (at%)	Co_3_O_4_ (at%)	CoO (at%)	Co(OH)_2_ (at%)	Co_2_O_3_ (at%)	Co_2_N_3_ (at%)
FeCoNiCr	0.00%	0.00%	0.67%	75.66%	23.67%	0.00%
FeCoNiCr_APPJ	0.00%	46.33%	21.25%	32.42%	0.00%	0.00%

**Table 6:** XPS Ni2p deconvoluted peak areal ratios from XPS spectra shown in Figure 10.

	Ni (at%)	NiO (at%)	Ni(OH)_2_ (at%)	Ni_2_O_3_ (at%)
FeCoNiCr	16.59%	44.36%	0.20%	38.85%
FeCoNiCr_APPJ	0.00%	0.00%	0.00%	100.00%

**Table 7:** XPS Cr2p deconvoluted peak areal ratios from XPS spectra shown in Figure 11.

	Cr (at%)	Cr2O3 (at%)	Cr(OH)3 (at%)
FeCoNiCr	13.22%	44.47%	42.32%
FeCoNiCr_APPJ	0.00%	45.13%	54.87%

## Supplementary Materials

The following are available online at https://www.mdpi.com/2073-4360/12/4/788/s1, Figure S1: Low-angle X-ray diffraction (XRD) of the FeCoNiCr alloy, Table S1: The crystallinity properties obtained from Figure S1, Figure S2: (**a**) Magnitude diagram and (**b**) phase diagram of the Bode plots, and (**c**) the Nyquist plots with EIS measurement of bare MEA for 12 h, Figure S3: (**a**) Magnitude diagram and (**b**) phase diagram of the Bode plots, and (**c**) the Nyquist plots with 12-h EIS measurement of APPJ-treated MEA for 12 h, Figure S4: (**a**) Magnitude diagram and (**b**) phase diagram of the Bode plots, and (**c**) the Nyquist plots with 12-h EIS measurement of OTS-coated MEA, Figure S5: (**a**) Magnitude diagram and (**b**) phase diagram of the Bode plots, and (**c**) the Nyquist plots with 12-h EIS measurement of OTS-coated APPJ-treated MEA. Figure S6: SEM images (magnification rate =5000X) of (**a**) bare, (**b**) APPJ-treated, (**c**) OTS-coated APPJ-treated, (**d**) OTS-coated MEAs.
