# Supplementary material for: Surface Modification of FeCoNiCr Medium-Entropy Alloy (MEA) Using Octadecyltrichlorosilane and Atmospheric-Pressure Plasma Jet
Source: Polymers (Basel). 2020 Apr 2;12(4):788. doi: 10.3390/polym12040788 (PMC7240591; doi:10.3390/polym12040788)
Supplement: Supplementary file 1 [file polymers-12-00788-s001.zip › polymers-759807-SI.docx]

Supplemental Material

| 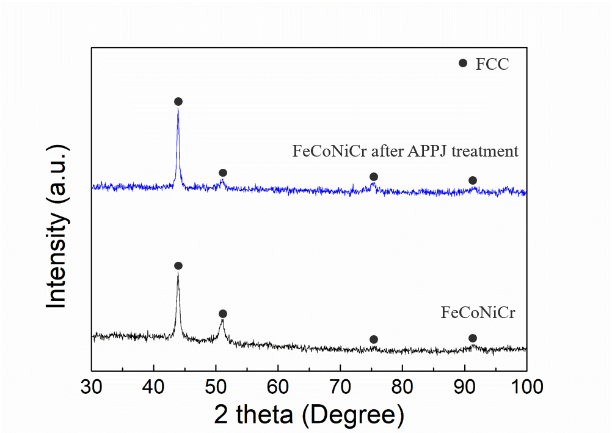 |
| --- |

**Figure S1.** Low-angle X-ray diffraction (XRD) of the FeCoNiCr alloy

**Table S1.** The crystallinity properties obtained from Figure S1.

|  | **FWHM** | **Kλ (nm)** | **β** | **2θ** | **D (nm)** |
| --- | --- | --- | --- | --- | --- |
| FeCoNiCr | 0.61708 | 0.15406 | 0.010770078 | 43.9 | 15.42 |
| FeCoNiCr_APPJ | 0.44391 | 0.15406 | 0.007747691 | 43.9 | 21.44 |

| 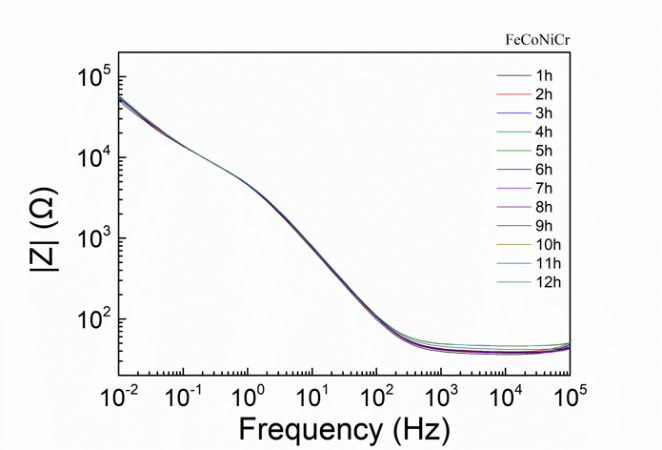  (**a**)  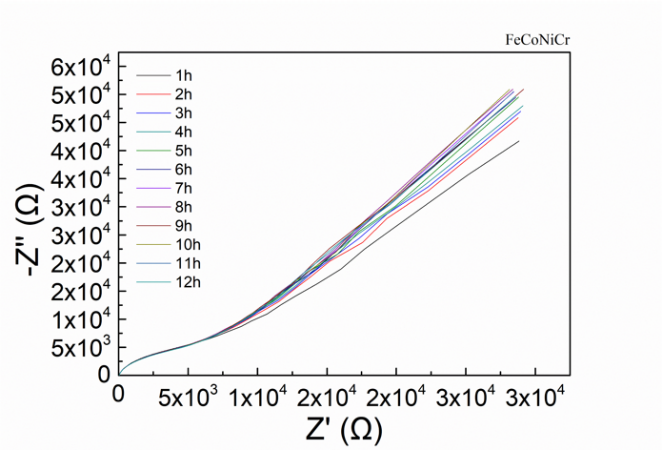  (**c**) | 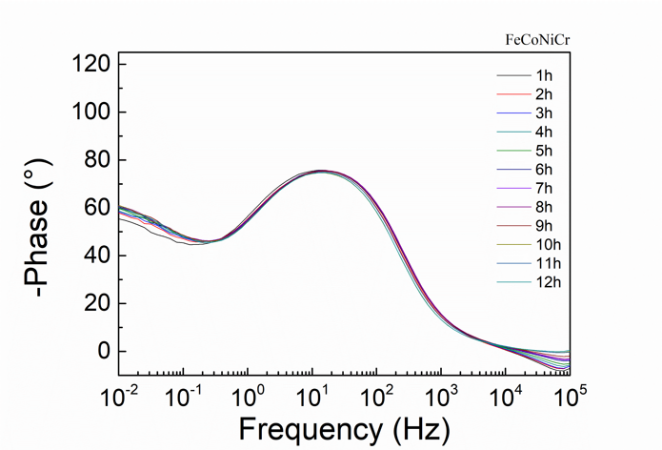  (**b**) |
| --- | --- |

**Figure S2.** (a) Magnitude diagram and (b) phase diagram of the Bode plots, and (c) the Nyquist plots with EIS measurement of bare MEA for 12 h.

| 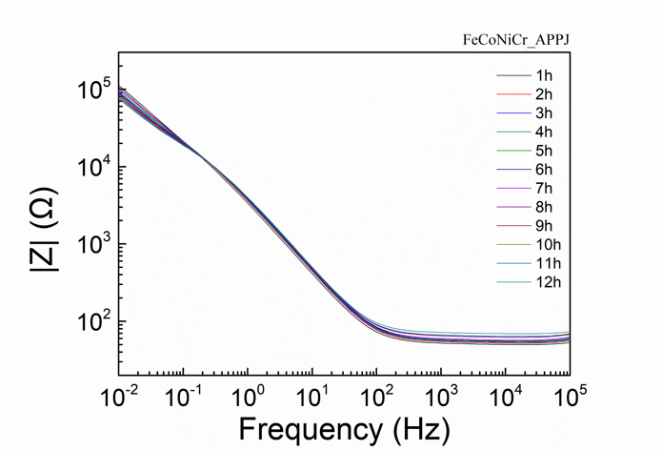  (**a**)  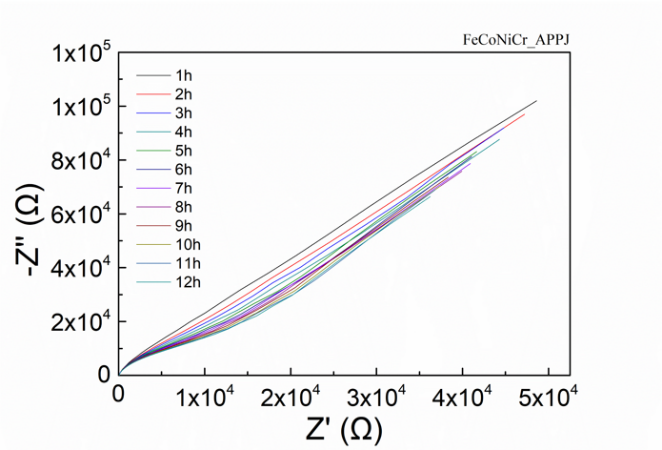  (**c**) | 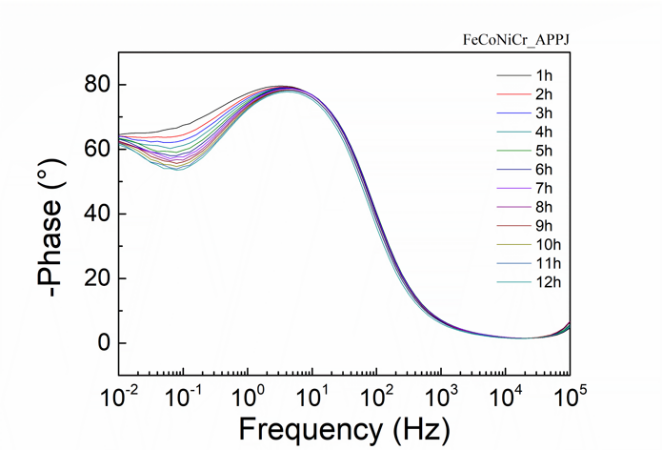  (**b**) |
| --- | --- |

**Figure S3.** (a) Magnitude diagram and (b) phase diagram of the Bode plots, and (c) the Nyquist plots with 12-h EIS measurement of APPJ-treated MEA for 12 h.

| 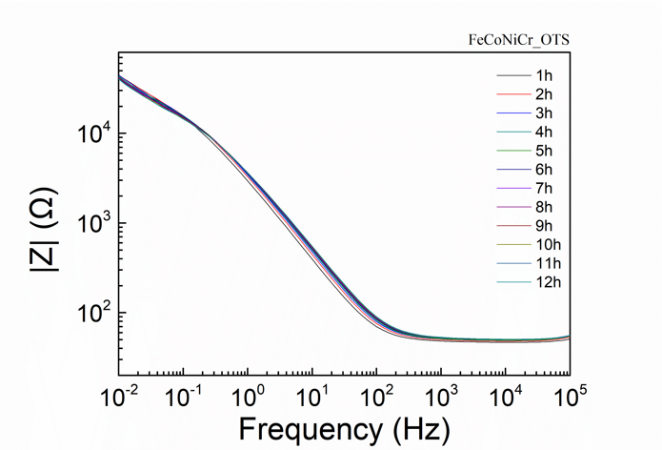  (**a**)  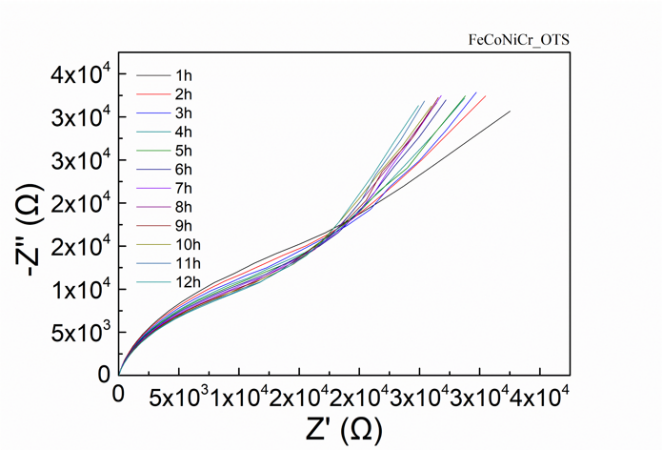  (**c**) | 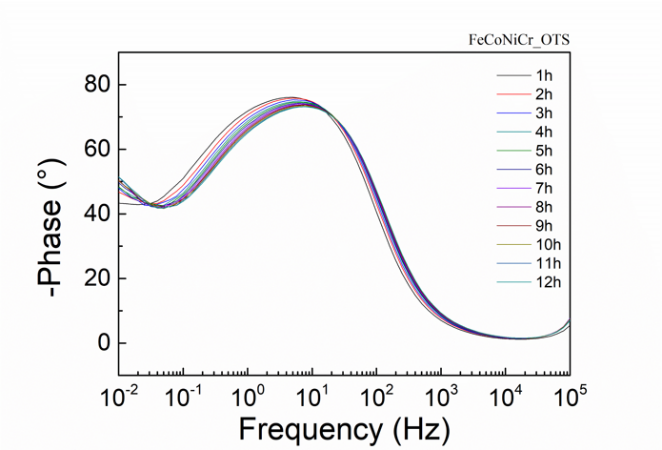  (**b**) |
| --- | --- |

**Figure S4.** (a) Magnitude diagram and (b) phase diagram of the Bode plots, and (c) the Nyquist plots with 12-h EIS measurement of OTS-coated MEA.

| 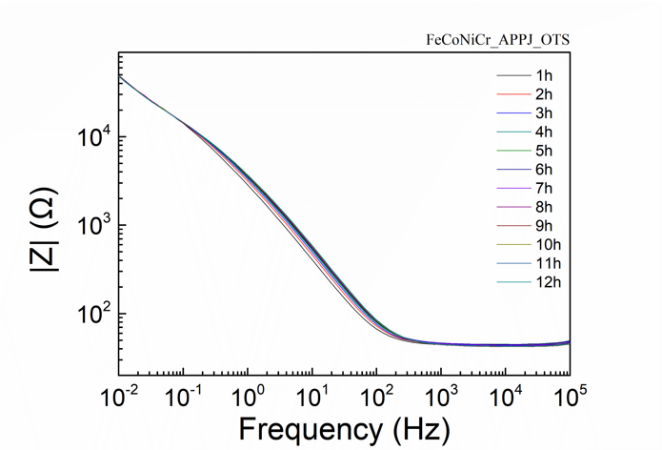  (**a**)  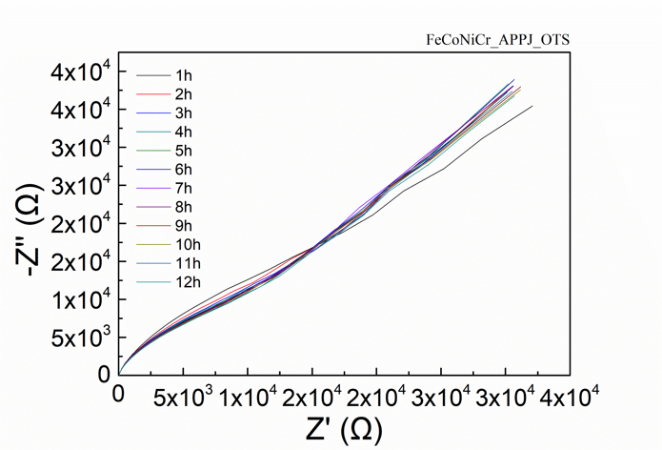  (**c**) | 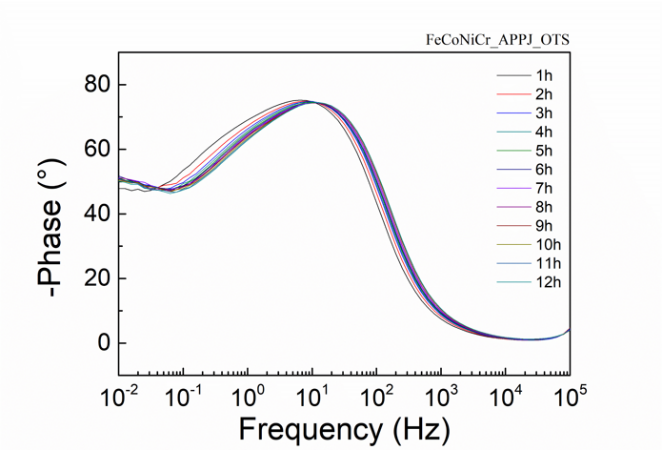  (**b**) |
| --- | --- |

**Figure S5.** (a) Magnitude diagram and (b) phase diagram of the Bode plots, and (c) the Nyquist plots with 12-h EIS measurement of OTS-coated APPJ-treated MEA.

| 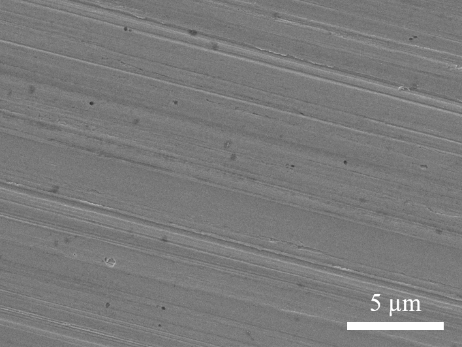  (**a**)  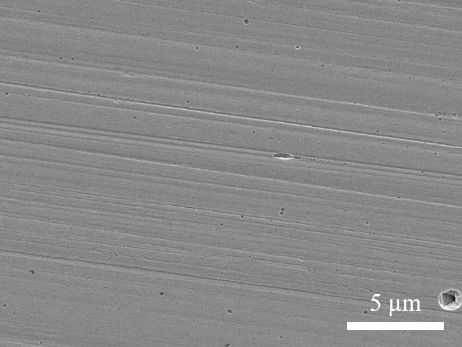  (**c**) | 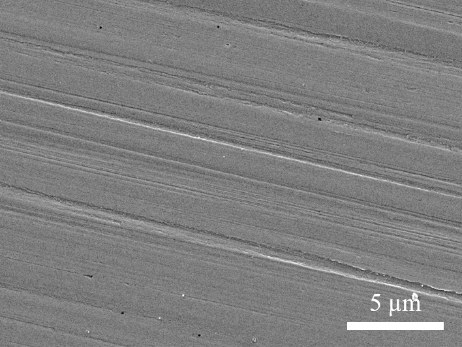  (**b**)  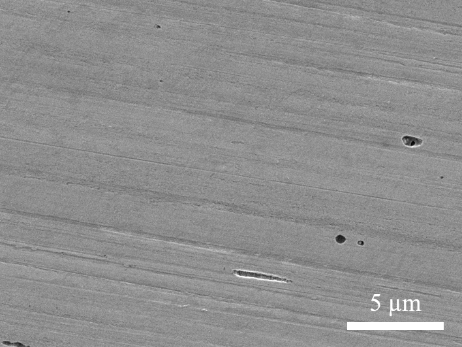  (d) |
| --- | --- |

**Figure S6.** SEM images (magnification rate = 5000X) of (a) bare, (b) APPJ-treated, (c) OTS-coated APPJ-treated, (d) OTS-coated MEAs.
